# Pharmacological plasticity—How do you hit a moving target?

**DOI:** 10.1002/prp2.532

**Published:** 2019-11-21

**Authors:** Michael J. Parnham, Gerd Geisslinger

**Affiliations:** ^1^ Fraunhofer Institute for Molecular Biology & Applied Ecology IME Branch for Translational Medicine and Pharmacology TMP Frankfurt am Main Germany; ^2^ Institute of Clinical Pharmacology J.W. Goethe University Frankfurt Germany

**Keywords:** chronobiology, drug metabolism and distribution, drug targets, Drug therapy, target variability

## Abstract

Paul Ehrlich's concept of the magic bullet, by which a *single drug* induces pharmacological effects by interacting with a *single receptor* has been a strong driving force in pharmacology for a century. It is continually thwarted, though, by the fact that the treated organism is highly dynamic and the target molecule(s) is (are) never static. In this article, we address some of the factors that modify and cause the mobility and plasticity of drug targets and their interactions with ligands and discuss how these can lead to unexpected (lack of) effects of drugs. These factors include genetic, epigenetic, and phenotypic variability, cellular plasticity, chronobiological rhythms, time, age and disease resolution, sex, drug metabolism, and distribution. We emphasize four existing approaches that can be taken, either singly or in combination, to try to minimize effects of pharmacological plasticity. These are firstly, to enhance specificity using target conditions close to those in diseases, secondly, by simultaneously or thirdly, sequentially aiming at multiple targets, and fourthly, in synchronization with concurrent dietary, psychological, training, and biorhythm‐synchronizing procedures to optimize drug therapy.

**Abbreviations**
CNR2: cannabinoid receptor 2
CNS: central nervous system
COX: cyclo‐oxygenase
DNA: deoxyribonucleic acid
EAE: experimental autoimmune encephalomyelitis
FCA: Freund's complete adjuvant
GLP‐1: glucagon‐like receptor‐1
GPCRs: G‐protein‐coupled receptors
HBV: hepatitis B virus
HDAC: histone deacetylase
HIV: human immunodeficiency virus
mTOR: mammalian target of rapamycin
NFκB: nuclear factor κB
NSAIDs: nonsteroidal anti‐inflammatory drugs
OATPs: organic anion‐transporting peptides
PG: prostaglandin
SCFA: short‐chain fatty acids
SFB: segmented filamentous bacteria
THP‐1: spontaneously immortalized monocyte‐like cell line, derived from the peripheral blood of a childhood case of acute monocytic leukemia
WHO: World Health Organization

## 1 INTRODUCTION

**Figure 1:**
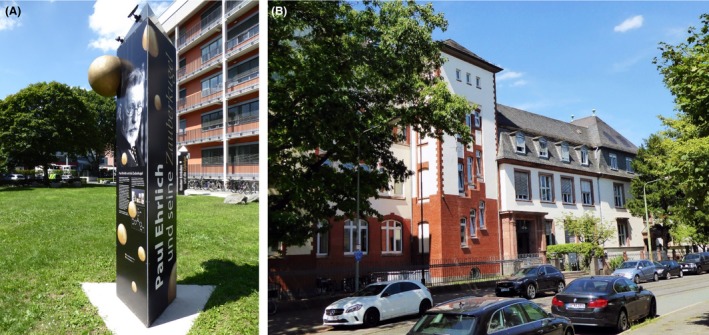
(A) Memorial to Paul Ehrlich and his theory of the magic bullet (*Zauberkugel*) erected in the University Hospital Frankfurt in 2015 on the 100th anniversary of his death (Photo: MJ Parnham) (B) The Georg‐Speyer‐House, Paul Ehrlich Strasse, Frankfurt am Main (Photo: MJ Parnham)

**Figure 2:**
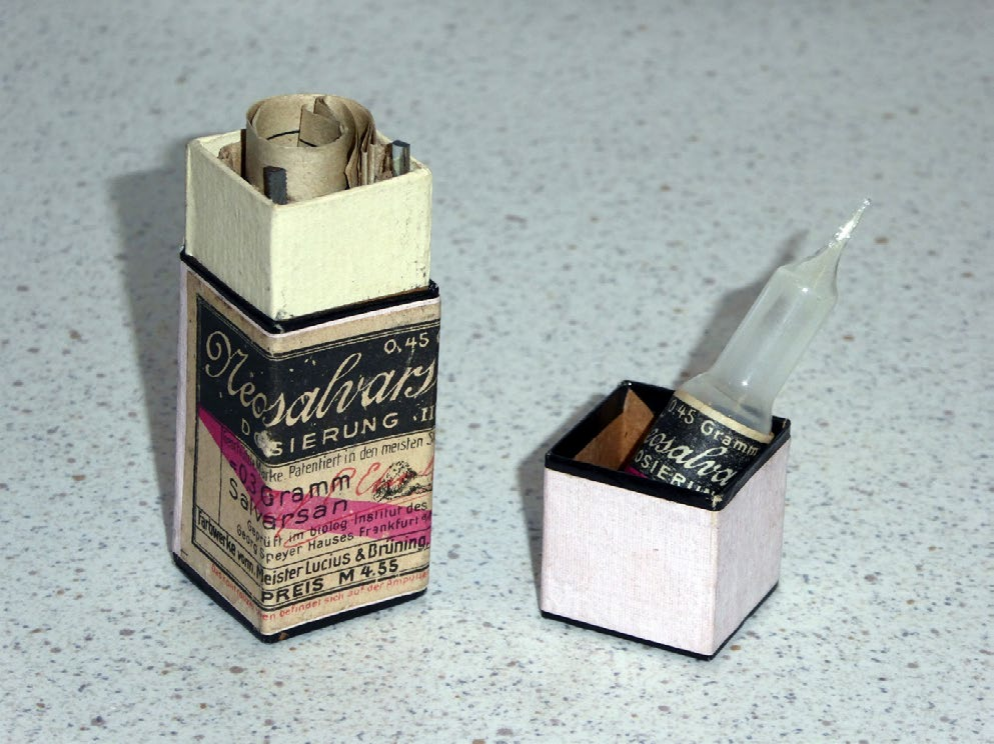
Original vial of Neosalvarsan Dosierung III (0.3g salvarsan) from Farbwerke vorm(alig) Meister Lucius & Brüning (later Hoechst AG), Hoechst am Main (Photo: MJParnham)

In August 2015, we commemorated in Frankfurt am Main, Germany, the 100th anniversary of the death of Paul Ehrlich, founder of the first Institute of Pharmacology and Experimental Therapeutics at Frankfurt University and the first Director of the Chemotherapeutic Research Institute, Georg‐Speyer‐House, now the home of the Institute for Tumor Biology and Experimental Therapy (Figure 1). Ehrlich was the first scientist to stain blood cells. He also identified the inheritance of immunity and propounded the side chain theory of antibody action, receiving the Nobel Prize for Medicine in 1908 together with Elie Metchnikoff.^1^ Ehrlich also discovered Salvarsan (Figure 2), an arsenic compound for the treatment of syphilis and showed that methylene blue could be used for the treatment of malaria. He suggested that a chemical compound could be used as a magic bullet (Zauberkugel) to selectively kill a disease‐causing organism, an idea which has been partially realized by the explosive development in recent years of monoclonal antibody therapeutics.^2^ But for most pharmacologists, the concept of the magic bullet remains an unattainable dream. Much as we should like to be able to hit a target with the accuracy and speed of a chemical bullet, the therapeutic target has an irritating habit of moving around.

Scientific research is an essentially rational occupation and phenomena are frequently reduced to the simplest system that can be studied. Look in any leading biomedical journal and the attractive colored diagrams of simplified interactive processes are among the first to catch the eye. We easily forget that living organisms are in a continual state of dynamic interactions and adaptations, biochemicals are being synthesized in bursts and dissipate, cells are growing and dying and remain almost continually on the move. Molecules both on the cell surface and in the cytoplasm, if visualized simultaneously, would probably look like a speeded‐up video of Time Square, Piccadilly Circus or the Place de l'Etoile in the rush hour. This mesmerizing mobility has to be taken much more into account when considering the effects of pharmacological agents, particularly if the dynamic homeostatic balance is to be maintained under treatment.

What do we mean by a moving target? The molecule aimed at with a drug may move location, from organ to organ or be transported within the body so that delivery of the drug is crucially important. The molecule may be present at a higher expression rate in some individuals or as a result of disease. In others it may be absent as a consequence of genetic differences such as single nucleotide polymorphisms so that the tested drug has no effect. As a corollary, the target itself may not move, but the treated organism can modify the drug, for instance by degrading the drug, meaning the “bullet” loses momentum and barely reaches the target. In addition, transport proteins (eg P‐glycoprotein) are expressed which carry the drug back outside the targeted cells. With some drugs, the effects observed may be unrelated to the target at which it is aimed, because unbeknown to the medical “hunter”, the “shot” has been sliced and a totally unexpected target has been hit, leading to so‐called “off‐target effects”. All these off‐target changes have a marked effect on the *precision* of the pharmaceutical bullet, since the reproducibility of the treatment is considerably reduced.^3^, ^4^ Alternatively, the target molecule can transform, not just in an oncological sense. It may occur in different isoforms or conformations, be changed or differentially expressed in response to cell stimuli or pathophysiological processes and can be modified over time in culture, with the time of day or as a result of aging. These changes in the nature of the target modify the *accuracy* of the drug treatment,^5^ so that what appears to be a true assessment of its efficacy in one study or population is no longer true under different conditions.

Positively, off‐target effects have contributed in part to the growing interest in repurposing of known drugs for novel therapeutic indications.^6^ But the outworking of a moving target becomes immediately obvious when our pharmacological bullet overshoots the target and hits something else and we end up with undesired toxicity.^7^ In the last few years, another aspect of this sort of collateral damage has been revealed by the fact that so many studies—many published in highly reputable journals—are poorly reproducible,^8^, ^9^ so the target does not seem to be in the same place in every lab! This may be due, among other things, to poor experimental design and methods, inadequate sampling, false use of statistics, lack of suitable controls or simply too much haste to get the data published. In this case, the set‐up of the target is incorrect and it becomes wobbly or falls over completely. A number of measures to counter this superficial science are being taken by various organizations, including the National Institutes of Health in the United States, the UK National Centre for the Replacement, Refinement, and Reduction of Animals in Research and the European College of Neuropsychopharmacology, as well as several leading journals.^9^, ^10^ It is hoped that this will enhance both robustness and reproducibility. Varied conditions or differences in gene expression in the organism or cells studied, the time of day of dosing or differences in the metabolic fate of the chemical agent, though, also contribute to contradictory findings.

**Figure 3:**
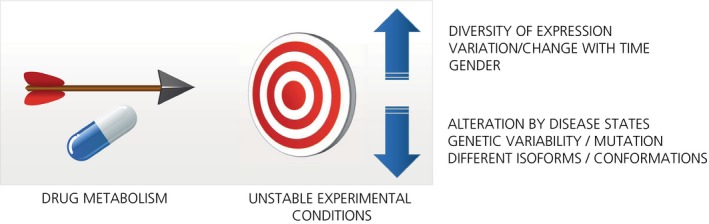
Factors contributing to pharmacological plasticity

Drawing to some extent on personal experience, particularly in the field of inflammation and (auto)immunity, we review here some of the reasons for the mobility of drug targets and their interactions with ligands (Figure 3) that result in pharmacological plasticity. We do not take into consideration the inherent chemical dynamics of target molecules themselves nor do we address neuronal plasticity. The CNS is inherently highly plastic. New neuronal connections and pathways are generated or lost in response to emotional experiences, environmental stress or learning, disease, trauma, or conditioning and this subject has been extensively addressed.^12^, ^13^ We do suggest, however, ways in which the drug target can be made to stand still or at least become easier to hit. Many of the approaches are already known. We emphasize the importance of taking most or all of the varied conditions into account, in a holistic manner, when developing drugs or explaining their effects in an attempt to tackle the “biological traffic melee” which most chronic diseases present.

## 2 GENETIC AND PHENOTYPIC VARIETY

One of the most important ways in which target molecules can alter their shape or form or even disappear is by changes in their gene expression.

### 2.1 Mutation

Gene mutation is one of the most widely appreciated sources of variability in response to drugs. It occurs relatively infrequently because of the effective deoxyribonucleic acid (DNA) repair mechanisms in most cells and competent immune surveillance, but under environmental and selection pressure, the mutation rate is increased. For immune defense, this can mean that epitope drift occurs, together with loss of antigens for host immune attack. Viruses are probably the champions at mutating^16^ in order to avoid destruction by vaccines or drugs. With global concern awakened by recent epidemics of influenza, Severe Acute Respiratory Syndrome, Ebola and Zika viruses, the need for effective antivirals has become more urgent. The most promising way forward with the most capricious of viruses, such as human immunodeficiency virus (HIV), appears to be to hit the virus at different sites with multivalent vaccines.^17^ This multi‐barreled approach has also proved effective in the drug therapy of HIV infection. With a combination of drugs, including non‐nucleotide and nucleotide reverse transcriptase inhibitors, protease inhibitors, and viral entry inhibitors, the introduction of Highly Active Anti‐Retroviral Therapy, composed of at least three different drugs, has effectively reduced the mutation rate and enabled many chronically HIV‐infected patients to live relatively normal lives.^18^ Such combination therapy directed at a specific viral target is also under extensive investigation for the therapy of other viral infections, including hepatitis B virus (HBV) infection, for which a four‐drug combination was recently marketed.^19^, ^20^ However, inclusion of the hepatitis C NS5B protein inhibitor, sovosbuvir, in antiviral drug combinations has dramatically enhanced success rates in the treatment of all genotypes of hepatitis C, indicating the value of aiming specifically at a crucial target.^21^

If the target is not specific and the use of the therapeutic armament is too indiscriminate, like a shotgun, the long‐term therapeutic outcome can be potentially worse than the disease originally targeted. This was the case in the 1960s, when the indiscriminate use of dichloro‐diphenyl‐trichloroethane (DDT), as highlighted in the milestone 1962 book “Silent Spring” by Rachel Carson, resulted in extensive environmental damage and spawned the ecology movement. In many ways, we are facing a similar situation with the indiscriminate use of antibiotics, particularly for nutritional livestock or for viral infections, which is actually promoting bacterial resistance mechanisms, such as drug efflux and methylation.^22^ Warnings about the global increase in bacterial resistance to antibiotics and the consequent loss of treatment options for many infections are now common and governments and industry have started joint efforts to combat the problem.^23^, ^24^ The situation highlights the importance of taking a multi‐target approach, but also increasing specificity and developing more effective and rapid diagnostic procedures to identify the potential etiological micro‐organism(s).

G‐protein‐coupled receptors (GPCRs) in cell membranes represent a major family of molecular targets for a variety of different drugs. Recently, it has been shown, on the basis of an analysis of UK National Health Service drug prescription and sales data from 68 496 individuals, that an average of 3% of these individuals carry at least one allele with a mutation at the active site of a GPCR drug target.^25^ For some GPCRs, the incidence of missense changes is much higher. Thus, over 86% of individuals carry at least one missense mutated allele at the active site of the cannabinoid receptor 2 (CNR2) and 69% in the glucagon like peptide 1 (GLP1) receptor, respectively. These two GPCRs are targets of the common antiemetic, nabilone, and several antidiabetic drugs such as exenatide, respectively. For these and other classes of drugs, the incidence of mutations suggests that there is an extensive propensity in the population for genetically induced, differential or even absent responses to drugs acting on GPCRs. The authors actually tested experimentally some variants of mu‐opioid and cholecystokinin‐A receptors and confirmed that different responses to actions at the variant receptors, compared to those on the wild‐type receptor, were obtained for a full agonist (morphine), a partial agonist (buprenorphine), an antagonist (naloxone) and the endogenous agonist endomorphine‐1.^25^

Together with infectious diseases and lifestyle disorders, cancer is a major global indication for drug development. Plastic changes in tumors have long stymied specific therapeutic targeting strategies. These changes include uncoupling of homeostatic regulation of growth in tumor cells, their resistance to drug therapy, for instance, by overexpressing efflux transporters such as P‐glycoprotein, and aggressive invasion of surrounding tissue. Although new approaches, such as kinase inhibition and immune checkpoint therapy are beginning to have an impact, use of cytotoxic drugs remains a mainstay of oncological treatment. 26 ^,^ 27 Here again, these drugs affect a wide spectrum of cellular growth processes. Adverse effects of cytotoxic drugs, particularly on the gastrointestinal system are pronounced, but more sinister long‐term genetic effects can arise. For instance, treatment of mice with the chemotherapeutic agents, cyclophosphamide, mitomycin C, or procarbazine caused apparent genome‐wide instability with significant elevation of simple tandem repeat mutation frequencies in the sperm and bone marrow of their offspring.^28^ Thus, intergenerational effects of anticancer therapy may potentially place the children of treated parents at risk, suggesting that a warning of potential effects on offspring should be given to patients of childbearing age to be treated with chemotherapy. As with infectious diseases, the collateral damage caused by nonspecific cytostatic therapy could well be a health threat to future generations and highly specific multi‐targeting could well be a better option. In fact, the most recent information‐based research suggests that a variety of different tumor types exhibit a recurrent regulatory structure consisting of functional master regulator proteins which are dysregulated in a posttranslational manner.^29^ Multiple targeting of this network of master regulator proteins, potentially starting also with screens in vitro, may be a way ahead for cancer therapy.

### 2.2 Heterogeneity and dynamic variability of biological systems

For a color blind marksman, the use of red or green in delineating a target would clearly present him with difficulties in aiming. The genetic variation in a target molecule presents a far more complex challenge to the pharmacologist. Under many experimental conditions, the use of hybridoma cell lines or inbred laboratory animal species goes some way to ensure that the genetic background of the cells or organisms is similar, wherever the experiment is performed. The expectation is that by ensuring that the genetic background remains reasonably constant, the cellular and physiological responses under standardized conditions will also remain relatively constant. Unfortunately, this is not always the case.

Even if the underlying genetic background is similar, what happens to gene expression when a different cell medium is used, the cell lines are taken after varying times of passaging or the incubation conditions used result in differing rates of growth? Many cell biologists are familiar with the fact that cell lines can become unresponsive due to some form of gene silencing when cultured for prolonged periods. But recent discoveries clearly show that cells respond very differently when the pH or oxygen concentration alters, as occurs, for instance during inflammation or ischemia. As a consequence, the effects of tested drugs change (www.ddw-online.com/therapeutics/p315005). In addition, cell stimuli are often grouped together by scientific investigators on the basis that they ultimately result in the generation of the same cell product with a similar outcome. For instance, prostaglandin (PG) E_2_ production by macrophages has been used for decades as an experimental readout for a response to an “inflammatory stimulus.”^30^ This is in keeping with the pathological situation, but PGE_2_ generation following stimulation of macrophages by antigen‐antibody complexes, calcium ionophore or phagocytosis occurs under the influence of different signaling pathways and from cells with different phenotypes. Most importantly, PGE_2_ also exerts anti‐inflammatory effects. These include suppression of phagocytosis and triggering of the resolution phase, when acute inflammation subsides. Thus, this lipid inhibits its own production via a negative feedback loop or by inducing generation of proresolving mediators and thereby modulates subsequent adaptive immunity.^31^, ^32^, ^33^ So the type of inflammatory stimulus, the time and inflammatory phase during which cells are collected, as well as the type of response measured can markedly alter the effect of the compound tested (see also section 5. Time, age, and the resolution of disease). The use of a defined stimulus to elicit a particular pharmacological or phenotypic response from a standardized cellular or biological test system would seem to be a solution to many of the issues outlined above. Across a variety of laboratories, responses to pharmacological intervention can then be compared much more easily. The problem is that Nature does not play fair and a disease syndrome can be the result of a whole range of different triggering agents. As far as possible, drug screening, therefore, should be carried out in a milieu that reflects the target disease environment.

Macrophages are remarkably plastic, depending on the stimulus applied and the expression of macrophage genes is highly susceptible to post‐translational epigenetic modifications, with silencing of some genes and activation of others.^34^, ^35^ Consequently, macrophages can express a wide range of phenotypes, from classical inflammatory M1 to anti‐inflammatory, alternatively activated M2 macrophages. Some researchers consider the cells to be in a continual dynamic state, altering their phenotype according to the conditions of the extracellular milieu.^36^ Moreover, the polarization of macrophages to a particular phenotype is under tight metabolic regulation, so changes in glucose, pH and other nutrients can change the genetic expression profile of the cells.^37^ This appears to be related to a “rewiring” of the citric acid (Krebs) cycle by inflammatory stimuli, simple products of the cycle such as citrate, succinate, and fumarate exerting remarkable changes in macrophage and dendritic cell function.^38^ We have also shown recently that the resting polarization state of primary human macrophages and the THP‐1 macrophage cell line differs, the latter being more polarized to an M1 state, with the relevant modifications in gene expression profile.^39^ Consequently, different forms of the same cell, in terms of cell lines and primary cells, particularly white blood cells, do not necessarily produce similar responses. To be able to gain a more accurate assessment of the effects of pharmacological agents on this type of cell, it is therefore, crucial to select a variety of cell phenotypes under different physiologically relevant stimuli, with evaluation of gene expression, cell signaling, and surface molecule expression changes, as well as the determination of secreted products. A variety of drugs have been reported to change the phenotype of macrophages, both in vitro and in vivo^40^, ^41^ so careful dissection of a spectrum of responses is needed to identify differences in pharmacological mechanisms.

### 2.3 Epigenetic changes

Gene regulation by epigenetic mechanisms, such as histone acetylation or methylation or DNA methylation, has been widely reported in many cells. Such mechanisms have been reported to play important roles in the etiology of cancer and have led to the introduction of the histone deacetylase (HDAC) inhibitors, vorinostat, romidepsin, belinostat, and panobinostat, as inhibitors of tumor proliferation in several types of cancer.^42^ However, these drugs are pan‐inhibitors of different HDAC types, each with different specificities and functions, so they have pronounced clinical side‐effects. Moreover, in addition to histones, other proteins that are subject to acetylation might also be affected by HDAC activity. Interestingly, it appears that concomitant inhibition of cancer‐associated inflammation with an inhibitor of the transcription factor, nuclear factor κB (NFκB), may act synergistically with HDAC inhibitors and reduce side‐effects—a further indication of the potential benefit of combined drug therapy of disease.^43^

Often the epigenetic modifications seen in healthy and transformed cells are studied experimentally only for a few hours or days. In this case, their effects should be viewed rather as cell signaling responses than as inheritable changes. Assessment of long‐term changes is a better way of assessing epigenetic inheritability. This can be done quite effectively in zebrafish or insects, because the generational turnover is much more rapid than in mammals.^44^, ^45^ Many drugs, particularly the anticancer agents discussed above, modify posttranslational changes. But other types of drugs, including analgesics, are able to modify epigenetic markers such as histone acetylation and DNA methylation.^46^, ^47^ Thus, the post‐translational silencing or activation of genes may be continued to subsequent generations. In this respect, a warning note is raised by the finding discussed earlier that treatment of male mice with standard chemotherapeutic drugs results in transgenerational instability in their offspring.^28^ Variability in natural populations is often driven by environmental or infection induced posttranslational genetic modifications and it is highly probable that drug treatment may need to be included among the modifying factors.^48^

## 3 AUTOIMMUNITY

Variability is an essential characteristic of the immune response, enabling adaptive immunity to be directed toward a vast repertoire of antigenic determinants. The expression of cell surface molecules, the cell phenotype, and subpopulations frequently change and the cells are continuously moving within the body, not only in blood and lymph fluid, but also across mucosal membranes and into and out of organs and tissues. In addition, though, a number of poorly considered factors also cause unexpected variation and jeopardize the investigation of autoimmune disease conditions. Taking our recurrent traffic metaphor, immunotherapeutics may be seen as traffic signals introduced to stop, slow down or redirect immune responses.

### 3.1 Models of autoimmunity

Sensitivity to the induction of experimental models of autoimmune diseases is highly susceptible to the genetic background of the strain of animals used, often resulting in differing phenotypes which respond differently to drugs. For instance, a widely used experimental model of multiple sclerosis is experimental autoimmune encephalomyelitis (EAE). This is commonly induced in C57BL/6 mice by immunological challenge with myelin oligodendrocyte glycoprotein, an important autoantigen in the CNS, administered in the immunological adjuvant, Freund's complete adjuvant (FCA, killed *Mycobacterium tuberculosis* in paraffin oil), with or without pertussis toxin to make the blood‐brain barrier permeable. Animals develop a progressive form of paralysis which can be scored.^49^ A variety of active compounds and marketed drugs has been developed on the basis of their activity in this model. However, only a minority of multiple sclerosis patients exhibit a chronic progressive form of the disease. The more common relapsing‐remitting form, occurring in 85% of patients, can be better reproduced in SJL mice after challenge with myelin proteolipid protein in FCA with pertussis toxin or in Dark‐Agouti rats with rat spinal cord homogenate in FCA. We have recently shown that even when assessing established, marketed drugs, not all EAE models show similar responsiveness to already approved treatments.^50^ However, even in models which show little responsiveness in terms of clinical score assessment, the concurrent use of more clinically relevant variables, such as behavior or social interaction, uncovered unexpected efficacy. Other authors have reported that sensitivity to mechanical pain in EAE also varies between SJL and C57BL/6 mice.^51^ The lesson drawn is that the genetic and phenotypic variety in experimental models can be taken into account partially using a range of genetically sensitive strains and a selection of translationally relevant assessment methods to generate an efficacy profile that provides a more precise picture of drug efficacy.

As with viral susceptibility to immune attack, epitope drift or genetic spreading also occurs during the development of autoimmunity and can complicate interpretation of drug effects. Consequently, the immune system begins to recognize antigenic molecules beyond those of the specific, external chemical determinants to which a specific immune response was initially induced.^52^ Not only does this lead to autoimmune tissue damage but also to the recruitment of a variety of different humoral, cytotoxic, and immunoregulatory mechanisms. At a later stage of many chronic autoimmune diseases it is, therefore, difficult to determine the basic underlying defect. Genetic and biomarker studies may highlight common factors in the disease, such as HLA‐DR4 variants or anti‐citrullinated protein antibodies in rheumatoid arthritis, which help to stratify patients for therapy,^53^ once again emphasizing the need for a range of disease assessments in evaluating effects of drug therapy. But epitope drift can also complicate the use of experimental models of autoimmunity. One of us (MJP) well remembers performing a series of studies years ago on the tissue injuring effects of lymph node lymphocytes from Lewis and Wistar rats with adjuvant‐induced arthritis,^54^, ^55^ during which the reactivity of the lymphocytes was gradually lost with time, presumably due to epitope drift, and the project had to be stopped. These observations are supported by results of a study by other authors on adjuvant arthritis induced in Sprague Dawley rats from two different vendors.^56^ The rats from the two sources varied in their susceptibility to arthritis, as well as in immune (various proinflammatory cytokines) and endocrine (plasma ACTH and corticosterone) responses. The authors suggested that different types of genetic drift in the two colonies were probably responsible, a possibility raised in a previous publication.^57^ It would be well worthwhile to carry out a comparative study between different labs of the range of antigenic reactivity of lymphocytes from animals in which the same initial stimulus is used to induce the autoimmune response. One would guess that this reactivity would differ considerably, indicating that the researchers are not necessarily assessing the same response.

### 3.2 Diet, microbiota, and immune response

A balanced diet is essential for metabolic and cardiovascular health, but variations in diet can also have a marked effect on (auto)immunity. Moreover, it is now clear that changes in the functions of T cells, macrophages and dendritic cells are closely related to their metabolic states.^38^, ^58^ Most laboratory animals are fed standard laboratory diets, so changes in immune responses are generally due to administered immunological stimuli, but this is not necessarily translatable to the clinical situation where dietary conditions vary considerably. Although diet can be controlled during phase 1 clinical trials in human volunteers, such dietary control is much less feasible in later stage trials. Intake of various types of fatty acids, trace elements and fruits rich in vitamins can all affect immune responses. For example, adequate zinc and selenium intake is necessary for the effective functioning of the immune system.^59^ Among other effects, zinc is important as a cofactor for T‐cell signaling, thereby facilitating differentiation and maturation of T lymphocytes, as well as promoting phagocyte and NK cell function.^59^ It is often used as an adjuvant treatment for infections and is recommended by the WHO for infectious diarrhea in children in developing countries.^60^, ^61^ Selenium is crucial for the function of the antioxidant glutathione peroxidase family of enzymes and other endogenous selenoproteins, such as thioredoxin reductases. Deficiency of selenium is associated with neutrophil and lymphocyte defects, probably due to inadequate protection of membranes from oxidative injury.^62^ Vitamin E also exerts a similar protective action on membrane integrity.^59^ These findings have provided considerable stimulus to the use of dietary supplements to strengthen the immune response. There is also increasing evidence that probiotics such as *Lactobacillus* can not only modify the constitution of the gut microbiome but also promote innate immunity. In this way, they also indirectly enhance adaptive immunity.^63^, ^64^, ^65^ Even in laboratory animals, though, dietary changes can occur unexpectedly. In one study in which one of us (MJP) was involved, eicosanoid metabolites, including PGE2, were determined over a period of several years in platelets and inflammatory exudates from different experiments on laboratory rats fed an essential fatty acid deficient diet. With time, the quantities of eicosanoids generated enigmatically increased.^66^ The source was eventually found to be an unannounced change made by the diet manufacturer in the source of the oil used to ensure essential fatty acid deficiency.

In the meantime, it has been well established that changing the type of dietary fatty acid can have marked effects on the structural type of eicosanoid generated. For example, increasing dietary intake of omega‐3 fatty acids, which occur in high quantities in fish, reduces the amounts and conversion of the n‐6 fatty acid arachidonic acid to metabolites such as PGE2 and leads to synthesis of metabolites that either have different or limited biological activities, as well as exerting inhibitory effects *pe se* on inflammatory mediator production.^59^, ^67^ Moreover, omega‐3 fatty acids modify the composition of the gut microbiome, promoting bacteria with a less proinflammatory profile.^68^ In terms of (auto)immunity, gut colonization with commensal segmented filamentous bacteria (SFB) plays a crucial role. SFB promote mucosal IgA production and the formation of Th17 effector cells in the gut Peyer's patches. This results in exacerbation of inflammation in murine models of colitis, autoimmune encephalomyelitis and arthritis, but protection from diabetes.^69^ The effects of the gut microbiome on drug metabolism and efficacy are considered in section 7.

Surprisingly, although the presence or absence of food is well‐known to affect oral drug absorption, few studies have been carried out to investigate effects of different dietary constituents on responses to drugs. Dietary supplementation with sodium selenite during pregnancy in the rat was reported to increase survival from embryotoxicity induced by concomitantly administered sodium salicylate, though the mechanism was unclear.^70^ Effects on reactive oxygen species seem likely. In nude mice with experimentally induced pulmonary tumors placed on a high fat (12% wt/wt linoleic acid) diet, co‐administration of the NSAID indomethacin was found to significantly reduce tumor growth.^71^ However, in a longitudinal clinical study in 906 patients with colorectal cancer, a positive interaction between low‐fat diet and aspirin administration on cancer incidence could not be observed.^72^ Thus, while changes in dietary constituents can markedly influence immune responses, much remains to be done to assess whether such variation in diet modifies drug efficacy. Perhaps such steps could be viewed as an attempt to modify the fuel used by the biological traffic to a more “environmentally friendly” form.

## 4 CHRONOBIOLOGY

The flexibility and mobility of molecules and cells certainly does not explain all types of pharmacological plasticity. Changes in the external environment, in addition to modifying the epigenetic profile, nutritional condition, and gut microbiome, can also modify targets and their responses to drugs. The modern propensity for travel, for instance, carries with it additional constraints. Most of us are aware of the physical challenges of intercontinental air travel: jetlag, sleeplessness and lack of concentration and difficulty in adjusting to seasonal and daylight alterations. The reason is that our whole organism, including individual cells, is regulated by body clocks and autoregulatory products of cellular clock genes which regulate transcription factors and control our daily circadian and seasonal rhythms.^73^ Jetlag results predominantly from light‐induced modification in the suprachiasmatic nucleus, the master body clock, which governs subsequent changes in the periphery. These include alterations in the secretion of melatonin from the pineal gland which govern the sleep/wake cycle.^74^ Light is, thus, widely viewed as a “time giver” or in German, *Zeitgeber*. Not only is our sleeping behavior regulated by the light‐induced release of the pineal hormone melatonin, but the courses of diseases can also be modulated by light.^75^

An example is the common symptom of rheumatic diseases, morning stiffness, resulting from disturbance in patients of the diurnal modulation of the hypothalamic‐pituitary‐adrenal axis and endogenous anti‐inflammatory cortisol levels, as seen also with corticosterone in experimental arthritis in rats.^76^ Cortisol levels are inappropriately low in patients in the early morning and are mirrored by changes in peripheral blood mononuclear cell counts and proinflammatory cytokines which have the opposite circadian cycle.^77^ Restoration of normal circadian rhythms may well be the reason why in years past, patients with rheumatic diseases improved considerably when sent to sanatoria in sunny countries and helps to explain some of the regenerative effects of sleep in various disorders. The expression of receptors for glucocorticoids in peripheral cells and tissues also varies with the circadian rhythm of the HPA‐axis, so that sensitivity of tissues to therapeutic corticosteroids also changes. Since receptor sensitivity is low in the morning and high in the evening, it has been proposed that this may be a basis for adjusting the timing of corticosteroid therapy to reduce side effects.^78^ Similar circadian rhythms have been observed in the efficacy and pharmacokinetics of NSAIDs, probably because of the changes in circulating leukocytes.^79^ Moreover, blood pressure is also under control of circadian rhythms and it has been proposed that antihypertensive therapies should be given at bedtime to achieve optimal effects.^80^

Pain also underlies a circadian rhythm and most importantly, melatonin itself is thought to exert analgesic activity.^81^ Similarly, respiratory disorders, allergic responses and gastrointestinal disturbances are subject to changes in light. There is growing evidence that in patients with severe sepsis, kept in an intensive care unit for days, the circadian rhythm is disturbed. Modification of the light and dark cycle by intensifying the contrast and minimizing light‐dark disruption or treating with melatonin can exert beneficial effects on the outcome of the condition.^75^, ^82^, ^83^ At least in immuno‐inflammatory disorders, it is worth pointing out that the cyclical circulating concentrations of melatonin, itself a potent antioxidant and anti‐inflammatory agent, may exert a direct effect on immunopathology.^84^ All these light‐induced variables markedly affect the sensitivity and responsiveness of molecular targets and alter their response to drug actions.

Such cyclic changes may not just be diurnal, occurring over a 24‐hour light‐dark period, but seasonal (circannual) changes arise as a result of shortening and lengthening of the daylight time. This is not of great significance in laboratory animals, as they are kept under controlled conditions. It is highly likely that the pharmacological responses to many drugs in humans, however, depend on both circadian and circannual rhythms. It was shown recently that 400 genes associated with the immune system in white blood cells and adipose tissue show very marked circannual variations.^85^ In Europe, the peak expression of anti‐inflammatory genes, such as the anti‐inflammatory circadian transcription factor, aryl hydrocarbon receptor nuclear translocator‐like, and the nadir of inflammatory genes such as interleukin‐6, occurs in July and the reverse in December. The opposite pattern is seen in Oceania.

At least in humans, introduction of a healthy diet, regular sleep, and exercise can go a long way toward synchronizing many biorhythms and stabilize drug targets. Interestingly, such modifications of lifestyle have clear effects on chromatin modifications which reflect the extent of biological aging.^86^ This finding provides further evidence for the possibility to modify with drugs, diet, or exercise the epigenetic processes that are associated with age‐induced deterioration or adverse mutation.

## 5 TIME, AGE, AND THE RESOLUTION OF DISEASE

Not only do underlying biorhythms alter the properties of drug targets, but physiological and pathological processes also change with time. Biomolecules, in particular proteins are very dynamic and interactive, alter their location and undergo modification.^87^ Their movement generally slows down with time and they become less flexible or dysfunctional, as with tau proteins and prions in neurodegenerative disorders and lipoproteins in atherosclerosis, predominantly due to increasing oxidative stress.^88^, ^89^ As time progresses, tachyphylaxis, the loss of drug efficacy, can occur probably because of protein receptor desensitization or because of cellular senescence, so that even if the target is hit, an effect is no longer observed.^90^, ^91^ Using our traffic metaphor, some of the traffic starts moving slowly or breaks down, causing increased congestion. Thus, the most obvious effect of time is seen in the effect of aging, a major issue in societies with an aging population.

Elderly people are far more susceptible to disease, partially because of the slowing and deterioration of normal physiology (especially of renal function), but also because of dysfunctional defense and repair mechanisms. There is a gradual decrease in the barrier function of the mucosa in the elderly with development of innate immune senescence, which affects all the cells of the innate immune system.^92^ Polypharmacy in these patients is very common and the heterogeneity of studies on drug use in this group of patients hinders the development of approaches toward risk reduction.^93^, ^94^ In a cross‐sectional analysis of community‐dispensed, prescribing data obtained for 310 000 adults over a period of 15 years in Tayside, Scotland, 81% of patients receiving ≥ 15 dispensed drugs per day experienced serious interactions compared with only 11% of those to whom two to four drugs were prescribed.^95^ Often, additional drugs are prescribed to combat the side‐effects of other previously prescribed drugs. This situation is akin to the example described above of taking a shotgun to hit a small target, resulting in unnecessary collateral damage. For the good of many elderly patients, a regular reassessment of the number and doses of drugs administered is highly advisable. The authors are personally aware of several elderly patients whose condition improved considerably when drug treatments were markedly reduced! Less is sometimes more. In fact, a recent report describes the improvement in glycemic control in diabetic patients following reduction of the complexity of their medication regimen.^96^ Nevertheless, the authors are well aware that in elderly patients with multiple morbidities, “appropriate polypharmacy” may be needed to treat these patients adequately.^97^ The problem is that there are only a couple of guidelines dealing with the situation of comorbidity. In most cases, in patients suffering concurrently from several diseases, it is inappropriate to simply combine the drugs that are recommended by the respective guidelines which cover only the individual diseases.

Most marketed drugs have been introduced because of a specific mechanism of action, often involving a single major target. During the development of the drug, the pathophysiological roles of the relatively novel target mechanism may be inadequately understood. Only later, often as a result of the clinical use of the drug, does the pathological role of the target mechanism in the disease process become clearer. This clarification also includes the time or phase of the disease process during which the target mechanism is most intimately involved and therefore, most susceptible to pharmacological intervention. Unfortunately, this improved understanding of the optimal timing for drug administration does not always lead to a modification of disease therapy since established drug use tends to be modified more as a result of limiting side‐effects than as a result of optimization of efficacy based on improved understanding of pharmacology. Two examples of the importance of timing for drug efficacy can be cited.

Acetylsalicylic acid or aspirin has been used for anti‐inflammatory, analgesic and antipyretic therapy for nearly 150 years and it is now being used as an antiplatelet and potentially anticancer agent. It has been estimated that 2000 tons of acetylsalicylic acid are synthesized annually, though in many cases, together with other nonsteroidal anti‐inflammatory drugs (NSAIDs), it is clearly overused. This is particularly the case in the elderly, as the many thousands of deaths due to drug‐induced gastrointestinal bleeding attest.^98^ What most patients and presumably prescribing physicians are unaware of is the fact that NSAIDs generally provide only symptomatic relief from pain and inflammation and have little effect on the time course of acute inflammation. This is certainly true of the common cold, a viral infection, in which only the inflammatory symptoms are reduced.^99^ In this case, only short‐term use of the NSAIDs is advisable to reduce initial inflammatory symptoms and avoid their potential inhibition of actively generated, endogenous proresolving lipid mediators.^100^

Drugs which do facilitate resolution of inflammation are the macrolide antibiotics such as azithromycin which promotes the generation of the proresolving macrophage M2 phenotype. Macrolides are known to be particularly effective in the treatment of respiratory infections such as community acquired pneumonia and acute exacerbations of chronic obstructive pulmonary disease, as well as the rare inflammatory lung disorder, diffuse panbronchiolitis. The macrolide antibiotics accumulate in leukocytes, causing an initial stimulation of neutrophil function and thus, antibacterial activity. Subsequently they promote the generation of the M2 macrophage phenotype and the resolution of inflammation.^41^ As a consequence, their antibacterial use, requiring only a few days administration, has been extended to more long‐term use in a limited number of inflammatory conditions. Macrolides without antibacterial activity are also being developed for their immunomodulatory properties.^101^, ^102^ Moreover, the capacity of azithromycin to promote the M2 macrophage phenotype has also been shown to be of potential therapeutic benefit in the treatment of cerebral ischemic injury.^103^ Recognition of the potential of a mechanism as a target for the drug treatment of a particular disease does not, therefore, mean it can be administered at any time to any patient with the disease. The timing and stage of the disease to be treated is also crucial for successful therapy.

The duration of drug action is a further factor in assessing the interaction with its target. We have avoided more than passing consideration in this article of intramolecular dynamics of drug target molecules, but it is worth mentioning one aspect of drug‐ligand binding that can affect duration of drug action. The drug‐target residence time model has gained increasing acceptance over the last decade in describing the interaction of a drug with its molecular target.^104^ While the affinity of a drug for its receptor has dominated theoretical considerations in the past, the new theory assesses drug‐receptor interactions in terms of the time for which the drug engages its receptor. The slower the drug dissociates from the target receptor, the longer its duration of action. Potentially, this would mean that retention of a drug at its site of action should prolong its effect. With azithromycin, this could indeed be the case, as it accumulates rapidly in leukocytes but it is only slowly released from the cells, allowing it time to exert proresolving, immunomodulatory effects.^105^ This pattern with azithromycin is also the same in fetal tracheal epithelial cell lines.^106^ It would definitely be of great interest—in particular for chronic disease therapy ‐ to compare drugs with moderate intrinsic activity at receptors but with prolonged duration of action with the efficacy of potent, short‐acting drugs with high intrinsic activity. There could be some surprises!

## 6 SEX

In the past, preclinical studies on drug candidates were performed exclusively on male animals, to avoid the “inconvenient” alterations in sensitivity resulting from endogenous hormonal changes. But it has now long been clear that female responses to drugs often vary from those in males. These differences start with sex differences in disease susceptibility, possibly in part because of sex chromosome linkage, but also due to hormonal and metabolic distinctions, physical constitution and gender‐specific lifestyles. This is obvious in autoimmune diseases which are often more prevalent in females than in males. Systemic lupus erythematosus and Sjögren's disease occur mainly in females, while systemic sclerosis is fourfold, rheumatoid arthritis two‐ to threefold and multiple sclerosis twofold more frequent in females than males.^107^, ^108^ Females also typically develop higher antibody responses and experience more adverse reactions to vaccination than males.^109^ Similar findings have recently been made in collagen type II arthritis in DA rats. In females, the ratios of CD4 and IL‐17‐producing T cells to Treg cells were raised and the production of Ig2a immunoglobulins increased in females in comparison to males.^110^ In contrast, sepsis is less pronounced in women^111^ and physiological resolution of acute vascular inflammation in humans appears to be more effective in women than in men.^112^ This suggests that the breakdown of such effective resolution mechanisms may account, at least partially, for the more severe sequellae of chronic inflammatory disorders in women.

Interestingly, it appears that effects of sex hormones on the neuroimmune system may also account for the well‐known higher incidence of chronic pain and increased sensitivity to pain in women.^113^ Schizophrenia, though, is more frequent in young males (2:1) than in females, whereas this ratio is reversed in adulthood, probably because of protective effects of estradiol.^114^ A sex difference has also been repeatedly reported in the incidence of and drug efficacy in depression in men and women, whereas sex differences have also been found in animal models of depression. Consequently, it is more relevant for subsequent clinical development, especially in terms of autoimmune and neurological diseases, to use female animals or at least animals of both sexes, in early compound testing.^115^ In fact, the US National Institutes of Health have recommended that the sex of animals and of cells be balanced for preclinical research studies.^116^ The US Federal Drug Administration encourages pharmaceutical and medical device companies to provide data from clinical trials derived from both men and women.^117^ This, however, means that the number of animals or human subjects to be included in trials is increased for statistical reasons, a fact that needs to be taken into account by local regulatory authorities.

Pharmacological actions of drugs are also subject to sex differences and some of these, particularly in relation to cardiovascular effects, have recently been reviewed.^118^ While this variability is partially genetically based, there are indications, for instance with regard to ß‐receptor sensitivity, that a sex difference in receptor sensitivity exists. The distinction between males and females is especially pronounced in pharmacokinetic processes, since the expression of many drug metabolizing enzymes and the occurrence of adverse drug effects is subject to modification by female sex hormones.^118^ The authors of the review emphasized that it is crucial to design clinical trials to be able to distinguish between responses based on sex and to clearly assess the confounding effects of sex hormones.

## 7 DRUG METABOLISM AND DISTRIBUTION

The most well‐established source of plasticity in responses to drugs and a major reason for drugs to miss their targets is their kinetic fate within the body. For instance, sex hormones affect not only the pH and motility of the gastric intestinal tract, but also the expression of oxidative metabolizing enzymes and membrane transporter proteins.^118^ More broadly, genetic polymorphisms in many drug metabolizing enzymes complicate considerably the determination of suitable dosing in clinical studies. Polymorphisms in oxidative metabolism by cytochrome P450 (CYP) are very well documented and are the basis for clear guidelines on the assessment of potential drug‐drug interactions in terms of metabolic interference between concomitant drug therapies.^94^, ^119^, ^120^ As considered earlier, such metabolic interactions become even more important in the elderly, in whom polypharmacy is common and rate of metabolism decreases, making interactions much more likely.^94^ Exercise and sport on the one hand, but factors such as smoking on the other hand, can have profound effects on the expression of metabolic enzymes. These effects include discrete modification of the methylation of the key metabolic regulatory enzyme adenosine monophosphate‐activated protein kinase in blood and skeletal muscle cells.^121^ Consequently, lifestyle changes may not only affect susceptibility to disease, as discussed above in relation to chronobiology, but also the metabolism of drugs.

The gut microbiome can exert profound effects on drug metabolism. Thus, metabolism by gut microbiota of uremic solutes, bile acids and steroid hormones can lead to modification of both phase I CYP‐mediated oxidative reactions, as well as phase II drug conjugating enzymes, such as glutathione‐S‐transferases or sulfotransferases, affecting the metabolism of a number of drugs.^122^ Conversely, bacterial enzymes can directly metabolize some drugs, thereby affecting their efficacy. Bacterial azoreductase in the colon, for instance, degrades sulfasalazine, a drug used in the inflammatory bowel diseases Crohn's and ulcerative colitis, to the active metabolites 5‐aminosalicylic acid (5‐ASA) and sulfapyridine. Nevertheless, in the rat 2,4,6‐trinitrobenzenesulfonic acid (TNBS)‐induced colitis model, sulfasalazine itself is able to restore the TNBS‐induced gut dysbiosis, as reflected by increasing counts of short‐chain fatty acid (SCFA)‐producing and lactic acid‐producing bacteria, as well as decreasing counts of proteobacteria, thereby ameliorating colitis.^123^ Enhancement of SCFA production by gut bacteria is also thought to be one of the mechanisms by which the anti‐diabetic type 2 drug, metformin, modifies glucose metabolism and cardiovascular responses.^124^ Thus, changes in the gut microbiome, caused by diet or lifestyle differences, can potentially alter the metabolism and thereby the efficacy of drugs, even in similar strains of rodents, since as we discussed previously, animals from different suppliers can differ in responses to xenobiotics.^56^ Indeed, an extensive sequence analysis of the gut microbiome in three different mouse strains from two suppliers, revealed considerable differences in bacterial composition between the same strains from different suppliers.^125^

Polymorphisms in drug transporter proteins are a more subtle source of concern, since these differences affect not only metabolic behavior of drugs but also their specific distribution to organs, tissues, and cells. On the one hand, efflux transport of drugs out of tumors and bacteria is a major reason for drug resistance,^126^, ^127^ necessitating continual development of new approaches to cancer and infections, preferably using the multitarget approach to minimize the potential for resistance.^126^, ^127^ On the other hand, while differences in drug transporters are known to affect hepatic transport and kidney excretion of drugs, it is also worthwhile noting that these transporter proteins can also affect drug transport into other tissues and organs. For instance, organic anion‐transporting polypeptide (OATPs) regulate intestinal absorption of macrolide antibiotics.^128^ OATP 1A2 is most highly expressed in the brain and appears to regulate drug transport across the blood‐brain barrier.^129^ Polymorphisms in OATPs, which have a wide spectrum of pharmacological substrates, are of crucial importance for the effects of drugs^130^ and could potentially affect CNS bioavailability. Screening of patients for OATP polymorphisms could potentially optimize therapy with the relevant drugs.

Pharmacovigilance is, thus, not just required to evaluate clinical reports of accidental or unexpected drug‐drug metabolic interactions, but should be performed proactively, recommending protective or screening measures to be taken based on new data from the literature and seeking to modify existing prescription behavior, as in the elderly.

A variety of technological approaches can be taken to prolong duration of drug action, particularly in their formulation and thus, to combat variation in drug metabolism. These include delaying absorption or using excipients which allow slow release of active ingredient from the formulated drug or hijacking transport mechanisms.^131^, ^132^ In this respect, an interesting new approach has recently appeared. Incorporation of deuterium (the heavy hydrogen atom) in place of hydrogen into a drug molecule creates stronger bonds with carbon and is widely used to synthesize reference compounds for drug analysis by mass spectrometry. The dopamine‐depleting drug, tetrabenazine has also long been used for the treatment of hyperkinesia, as well as Huntingdon's disease. By introducing deuterium into the tetrabenazine molecule, it was shown that the metabolism of the molecule at that site can be slowed down, and both peak plasma concentrations and potential side‐effects reduced.^133^, ^134^

## 8 CONCLUSIONS AND PERSPECTIVES

In view of the immense variability and plasticity of the mammalian organism in response to pharmacological agents, it is not entirely surprising that drugs do not always provide the benefit we expect. In fact, it has to be admitted that even when a new drug is marketed we know relatively little about its efficacy in a large population and subsequently, many drugs are prescribed too often for unsuitable conditions and patients are too willing to take medicine for conditions which may not require such treatment. Aside from this, nonadherence is also a big problem.

Apart from highly specific drugs such as monoclonal antibodies, it may be worth reconsidering the rational approach to drug therapy, namely of using a single specific drug to hit a single target, like Ehrlich's magic bullet. Rather, we should consider the multiple factors which make the target difficult to hit and try using a progressive, multifactorial, multitargeting approach. There are enough examples of this technique in the natural world. When lionesses hunt a fast moving and gyrating antelope, they do so in a group. This initially reduces the space within which the antelope can move and then one of the attackers may be able to injure and slow down the prey before the others come in for the kill. The same approach is used in military actions, to slow down or damage a superior opponent and thereby, it becomes easier to destroy. To use the metaphor we started with in the introduction, the sometimes crazy movement of cars at a major road intersection is best controlled by forcing the cars into specific lanes and introducing co‐ordinated traffic lights. The potential for traffic jams is markedly reduced.

In pharmacological terms, this can be achieved by the co‐ordinated use of different drugs. A comparable concept has been proposed in relation to drug doses used for the treatment of disease.^135^, ^136^ The authors, for instance, suggested that a combination of low doses of anti‐inflammatory analgesic drugs acting at different stages in the generation and action of PGE_2_ [on release of substrate by phospholipase A_2_, metabolism of arachidonic acid by cyclo‐oxygenase and microsomal PGE synthase and finally action of PGE_2_ on its different receptors] could be used. In this way, the adverse gastric and cardiovascular effects arising from pronounced inhibition of cyclo‐oxygenase with NSAIDs could be markedly reduced. Alternatively, an immune reaction or neurological deficiency could potentially be regulated by light or melatonin to make the final target more accessible to treatment. The growth of a dysregulated cancer or other cell could be slowed so that more time is available to target a more specific mechanism. In this way, the serial use of two to three pharmacological agents, preferably with long drug‐target residence times, would probably have greater efficacy than a single potent agent that only proves to be effective in a limited number of patients because of the inherent instability of the target mechanism. This approach is already taken to good effect in the treatment of hypertension in which a diuretic, for instance, is combined with a sartan and/or a calcium antagonist to consistently decrease blood pressure.^137^

**Figure 4:**
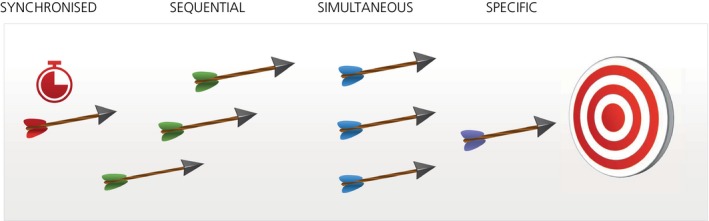
Proposed drug treatment measures to reduce pharmacological plasticity

We emphasize four already existing approaches (Figure 4) that can be taken either singly or in combination, to try to minimize effects of pharmacological plasticity:

*Specific*—whether aiming a drug at a single or multiple targets, the aim should be precise, providing a greater chance of avoiding off‐target effects. Despite the long history of drug targeting and years of improvements, there is still room to enhance chemical specificity at the drug design stage. Intriguingly, recent detailed docking studies with over 3 million compounds revealed it is possible to bias the effect of a compound acting on a single receptor toward one or the other downstream signaling processes. Docking to the μ‐opioid‐receptor led to the identification of a compound which preferentially activated signaling through the G protein Gi, with clear analgesic activity, avoiding signaling through the β‐arrestin pathway, associated with the side‐effects of respiratory depression, constipation, and tolerance.^138^ In view of the much increased understanding of signaling pathways, this might be a promising approach.

Screening conditions, in addition, should reflect, as closely as possible, those occurring in the immediate environment of the putative disease target. A recent review has drawn up a generic set of principles—including genomic context, cellular heterogeneity, incubation conditions and relevant stimuli—by which a suitable in vitro screening cell system can be established.^139^ Even repurposing of known drugs for new indications can be more specific. In this context, a set of guidelines has been proposed to facilitate in vitro test selection for kinase inhibitors—which have a variety of off‐target effects—in order to define potentially therapeutically beneficial off‐target pharmacological effects.^140^ Stratification of animal and clinical systems, based on biomarkers or genetic clustering, as well as age, sex, and disease stage, even diet, with consideration of circadian and circannual rhythms, should also improve specificity. To gain a hold on some of these less easily controllable variables, it is essential to make effective use of reference compounds. This involves checking whether their responses remain the same under all conditions and whether the pharmacological response from the test situation is as expected. Since the reference compound may also have some off‐target effects, a biomarker of its mechanism of action should be included.

*Simultaneous*—multitargeting several mechanisms at once. Currently, this usually involves treating diseases with multiple drugs. We have already seen how in the elderly this can cause problems. It is preferable if individual drugs can be developed with multiple relevant actions. Recent studies suggest that single small molecule compounds may be developed to hit several targets simultaneously. Thus, glitazone compounds have been described recently for metabolic disorders with partial PPARγ agonist, potent COX‐2 antagonist (nanomolar IC50 values) and moderate 15‐LOX inhibitor (micromolar IC50 values) activities. These compounds were anti‐inflammatory on macrophages in vitro and in acute inflammation in vivo.^141^ Whether this activity requires all mechanisms of action or can be attributed to just one or two of them, remains to be established. Other approaches to multi‐targeting include network targeting, as with optogenetics in which neuronal circuits are modulated by marking of specific genes with rhodopsin which responds to light,^142^ or with phenotypic assays which use functional readouts as opposed to single target‐based assays. Considerable interest has arisen in the use of phenotype assays, both in primary cellular or stem cell systems in vitro and in animal models of disease.^143^, ^144^ For instance, it was reported that NSI‐189, a benzylpiperizine‐aminiopyridine that stimulates general neurogenesis of human hippocampus‐derived neural stem cells in vitro, is able to exert multidomain effects on cognition and depression, suggesting that phenotypic multitargeting may also be viable for CNS disorders.^145^ The challenge is to identify, possibly by reverse pharmacology through extrapolation backwards from the clinical condition, a phenotype or set of symptoms to use as potential functional targets for initial screening. Subsequently, when an active hit or lead compound is identified, target deconvolution has to be carried out to investigate potential mechanism(s) of action. Recently, proposals have been made for establishing a “chain of translatability,” starting with the identification of a disease‐associated molecular characteristic or signature such as a disease‐associated gene expression profile.^146^ This is then succeeded by cellular models which aim to reconstruct a cellular phenotype as similar as possible to that of the disease condition and subsequently, use of relevant animal models of disease.

Alternatively, a wide range of assays and readouts can be used and a systems biology approach taken to assess the data for patterns. This approach is also taken in our laboratories, using different test models with a range of functional, molecular, biochemical, and imaging methods, at various stages of compound testing, to reflect the clinical conditions.^50^, ^147^, ^148^ Where the target consists of a complex of interacting mechanisms, as in pain, a computational functional genomics‐based approach can help to improve accuracy, clarifying both the specific targets to aim at for defined pain types and also offering insight into the target mechanisms for pharmacological modification either by individual drugs or a combination of therapeutic agents.^149^

*Sequential*—slowing down the target, before moving in to the hit/kill, by aiming sequentially at one or more targets upstream and then one or more targets downstream, using single drugs with multiple actions or a combination of drugs with varied actions. This is illustrated by the long‐established combination in a single dosage form of the antibiotics sulfamethoxazole, an inhibitor of dihydropteroate synthetase, and trimethoprim, an inhibitor of dihydrofolate reductase. Together they act synergistically to inhibit the bacterial synthesis of tetrahydrofolic acid. A further example of this approach is the sequential combination of drugs acting at different stages of cell signal pathways. Several growth factors act through the phosphoinositol‐3‐kinase/protein kinase B pathway which is crucial for cell proliferation and angiogenesis. A downstream effector of this pathway is the regulatory protein, mammalian target of rapamycin (mTOR). Addition of the anticancer drug, docetaxol to adenocarcinoma cells in vitro for 24 hours, followed by the mTOR inhibitor, temsirolimus was highly synergistic in suppressing phosphorylation of mTOR as well as in suppressing proliferation in lung cancer cell lines.^150^ The authors proposed that this sequential combination may be effective in overcoming resistance of tumors to mTOR inhibitors. Using a similar rationale, a phase 2 study was carried out in which the tubulin polymerization inhibitor, BNC105P was administered together with the mTOR inhibitor, everolimus, to patients with metastatic renal cell carcinoma who were refractory to tyrosine kinase inhibitors.^151^ While the primary endpoint was not reached, analysis of biomarkers suggested that further studies are warranted.

In chronic diseases, inhibition of epigenetic changes with the resulting slowing of the chromatin aging process may soon be possible, making patients more amenable to other types of therapy.

*Synchronized*—using concurrent dietary, psychological, training, and biorhythm‐synchronizing procedures to optimize drug therapy, but taking into account (and standardizing) possible changes in response with time and age. A wide variety of different pharmacological, physical, nutritional, and other procedures are used in the hospital setting to treat the ill patient. However, a therapeutic program synchronized for the various factors we have discussed is rare. Considerable potential appears to exist in modifying the gut microbiota to improve drug efficacy, as discussed previously in relation to drug metabolism. Thus, by modulating the gut bacterial population there is increasing evidence that the efficacy of current anticancer chemotherapeutics can be enhanced and their toxicity reduced,^152^ perhaps by simple measures such as regular use of probiotics. Obviously, in the treatment of all human diseases, drug therapy is usually just one of the measures taken to improve the health of the patient. But the optimal conditions for implementing supportive measures are not often assessed, certainly not in preclinical investigations. For instance, the efficacy of the appetite suppressant, diethylpropion, used clinically to cause short‐term weight loss, was greater when given to rats at night when their activity was greatest and when the animals were also placed on high fat dietary restriction.^153^ In many chronic diseases, muscle loss occurs and general metabolic deficiencies arise. In chronic obstructive pulmonary disease, for instance, there is increasing evidence that combined nutritional and exercise interventions can be effectively used in combination with drug therapy.^154^ In contrast, in the treatment of cancer, caloric restriction, as with ketogenic diets, has been found to both enhance drug efficacy and reduce toxicity.^155^ Measurements taken during such synchronized treatment studies should be made at set times and seasons, allowing for the fact that the duration of action of some drugs is long. Such synchronization of drug therapy with other factors will undoubtedly become possible in the near future, as the combined use of the 4Ds, drugs, diagnostics, devices and (big) data, becomes increasingly possible.^156^ In any case, even with established drugs, it is crucial to be vigilant and to be aware of new findings that can impact the way in which drugs are prescribed and used.

## DISCLOSURES

MJP has been a consultant for Leo Pharma and Xellia Pharmaceuticals and was previously an employee of GlaxoSmithKline. GG was a member of the IMI (Innovative Medicine Initiative of the EU) EuroPain collaboration, in which the following industry members are represented: Astra Zeneca, Boehringer Ingelheim, Eli Lilly, Esteve, Gruenenthal, Pfizer, UCB Pharma and Sanofi Aventis. GG has received honoraria as a speaker from Gruenenthal, Mundipharma, and Pfizer. He is a consultant for Abbvie. He has received research funding in the form of a grant from Mundipharma.

## AUTHOR CONTRIBUTIONS

Both authors were involved in drafting the manuscript or revising it critically for important intellectual content, have given final approval of the version to be published and are accountable for all aspects of the work.

**DATA AVAILABILITY STATEMENT**

Data used were obtained from secondary sources (EndNote, RRID:SCR_014001; Europe PubMed Central, RRID:SCR_005901; Google, RRID:SCR_017097) or represent information otherwise known personally to one or other of the authors, which is cited where possible. A link to a data repository is thus not relevant.
